# Effects of Robot Facial Characteristics and Gender in Persuasive Human-Robot Interaction

**DOI:** 10.3389/frobt.2018.00073

**Published:** 2018-06-21

**Authors:** Aimi S. Ghazali, Jaap Ham, Emilia I. Barakova, Panos Markopoulos

**Affiliations:** ^1^Department of Industrial Design, Eindhoven University of Technology, Eindhoven, Netherlands; ^2^Department of Mechatronics Engineering, International Islamic University Malaysia, Selayang, Malaysia; ^3^Department of Industrial Engineering and Innovation Sciences, Eindhoven University of Technology, Eindhoven, Netherlands

**Keywords:** trust, psychological reactance, facial characteristics, gender similarity, persuasion, human-robot interaction

## Abstract

The growing interest in social robotics makes it relevant to examine the potential of robots as persuasive agents and, more specifically, to examine how robot characteristics influence the way people experience such interactions and comply with the persuasive attempts by robots. The purpose of this research is to identify how the (ostensible) gender and the facial characteristics of a robot influence the extent to which people trust it and the psychological reactance they experience from its persuasive attempts. This paper reports a laboratory study where SociBot™, a robot capable of displaying different faces and dynamic social cues, delivered persuasive messages to participants while playing a game. In-game choice behavior was logged, and trust and reactance toward the advisor were measured using questionnaires. Results show that a robotic advisor with upturned eyebrows and lips (features that people tend to trust more in humans) is more persuasive, evokes more trust, and less psychological reactance compared to one displaying eyebrows pointing down and lips curled downwards at the edges (facial characteristics typically not trusted in humans). Gender of the robot did not affect trust, but participants experienced higher psychological reactance when interacting with a robot of the opposite gender. Remarkably, mediation analysis showed that liking of the robot fully mediates the influence of facial characteristics on trusting beliefs and psychological reactance. Also, psychological reactance was a strong and reliable predictor of trusting beliefs but not of trusting behavior. These results suggest robots that are intended to influence human behavior should be designed to have facial characteristics we trust in humans and could be personalized to have the same gender as the user. Furthermore, personalization and adaptation techniques designed to make people like the robot more may help ensure they will also trust the robot.

## Introduction

Social robotics is a research domain that focuses on the design of robots which socially interact with humans (Dautenhahn, 1994). Social robots show (some) human characteristics like using several verbal and non-verbal cues to express the robot's emotions and intentions (Breazeal, 2003; Weber, 2005; Hegel et al., 2009). Interest in social robots can be traced back to the mid-1900s (Goodrich and Schultz, 2007). Researchers have been exploring various roles that robots can play in a human-robot relationship. For example, researchers have developed social robots to mediate between Parkinson disease patients and their caregivers (Shim and Arkin, 2017), to promote collaboration and measure engagement between children with autism (Rudovic et al., 2017) and their siblings (Huskens et al., 2015), and as a tutor in learning applications (Gordon et al., 2016).

Similar to human-human relationships, evidence suggests that trust in the robotic interaction partner is crucial for developing human-robot relationships. Humans should feel safe to rely on social robots for physical or even emotional support (Rotter, 1967). Earlier research (Hancock et al., 2011) suggests that robot-related factors (such as the robot's performance), human-related factors (like personality traits of the human), and environmental factors (for instance the complexity of the task assigned) are crucial for developing trust in human-robot interaction. A meta-analysis by Hancock et al. (2011) concluded that robot characteristics are also instrumental in developing trust for human-robot interaction.

There have been a few attempts to endow robots with human-like features so that humans will find it easier to trust them. These include matching human likeness (Mathur and Reichling, 2016), behavior (Goetz et al., 2003), head movement and facial characteristics (gaze and eyelid movements) (Lee and Breazeal, 2010), and gestures (Tang et al., 2014). An earlier study (Verberne et al., 2015) showed that a significant characteristic that influences user trust is the similarity (looks, acts, and thinks) between the user and an artificial agent (Siegel et al., 2009). This research (Verberne et al., 2015) used the trust game concept (see also de Vries, 2004) to measure trust that the participants have in their (artificial) interaction partner. In this trust game, participants can allocated resources to their (artificial) interaction partner, which the game will double if the interaction partner collaborates, thereby giving a quite direct, behavioral measure of trust in that interaction partner.

A salient characteristic that also can be similar to the user is the robot's ostensible gender (for brevity we refer to it simply as gender in the remainder of this article). To date, only a few studies have investigated how the robot's gender influences trust and these studies have produced mixed results (Powers et al., 2005; Crowelly et al., 2009; Siegel et al., 2009; Eyssel and Hegel, 2012; Eyssel et al., 2012). Some earlier studies suggests that similarity between a robot's and a user (Verberne, 2015) especially similarity in terms of gender (Eyssel and Hegel, 2012) might increase the user's trust. Another experimental study (see Siegel et al., 2009) found that both men and women trust robots of the opposite gender more than robots of the same gender.

Robotics researchers have examined several approaches to encourage the attribution of gender to a robot so that people would perceive it more positively. For instance, male robots were given short hair and female robots long hair in evaluating gender-stereotyping tasks like monitoring technical devices and childcare in a study by Eyssel and Hegel (2012). Other research used robots with pre-recorded masculine or feminine voices in donation tasks (Siegel et al., 2009), or utilized a conversational robot that had gray vs. pink lips in discussions about dating norms (Powers et al., 2005). In a between subjects design study, Crowelly et al. (2009) used synthetic voices (male vs. female voices) and gender-specific names (“Rudy” for male robots vs. “Mary” for female robots) to manipulate user perceptions of robot gender. Based on the outcomes and research methodology developed in earlier studies (Siegel et al., 2009; Eyssel and Hegel, 2012; Alexander et al., 2014; Verberne et al., 2015; Jung et al., 2016), this article reports an experiment that examines the influence of the robot's gender on trust.

Trust toward the robot may also be influenced by its facial characteristics. It is well-known that humans make social judgments about other people's faces and similar reactions have been observed toward artificial agents. Earlier research suggests that the level of trust toward a social agent depends on various aspects, for example, its level of embodiment (robot, avatar, or a picture) (Rae et al., 2013), its ability to display social cues (Ruhland et al., 2015; Xin et al., 2016), and its appearance (Zlotowski et al., 2016). Mathur and Reichling (2016) found that the trustworthiness of a robot varies with the likeness of the robot's face to a human following a general pattern was known as the “uncanny valley” (see Mori, 1970): trustworthiness, in this case, does not increase linearly with human likeness but drops when the agent is very realistic but not yet perfectly human-like.

Todorov et al. (2008, 2015) examined how facial characteristics of a social agent can influence user's trust. They generated pictures of unfamiliar faces to display facial characteristics representing three levels of trust: most trustworthy, neutral, and least trustworthy (Todorov and Oosterhof, 2011). The generation of facial characteristics was evaluated on the basis of functional Magnetic Resonance Imaging (fMRI) of participants. Todorov et al. (2008, 2015) concluded that humans perceived upturned eyebrows and lips as the most trustworthy facial characteristic, while the least trustworthy face is the one with eyebrows pointing down and lips curled down at the edges. However, these results are still tentative, since facial characteristics were only represented in two-dimensional images, and have not yet been tested with an embodied agent or a robot. In addition, scholars like Vidotto et al. (2012) and McKnight et al. (1998) remark that there are different conceptions of trust toward interaction partners such as trusting beliefs and trusting behaviors. However, Todorov et al. (2008) did not specify which type of trust was generated from manipulating these facial characteristics. Furthermore, Todorov et al. (2008) only assessed first impressions toward the appearance of those characters and their study participants did not interact with the characters.

Trust is one of several social responses to robots that have been assessed earlierly in human-robot interaction. Another example pertains to positive and negative impressions people have of the robots. Positive impressions include social engagement with the robot (Moshkina et al., 2014), the effectiveness of the social actor in delivering messages (Katevas et al., 2015), the degree to which people perceive the robot as an intelligent agent (Talamas et al., 2016)and the anthropomorphism value for social acceptability (de Graaf et al., 2015). In contrast, several questionnaires were developed to measure negative attitudes (Tatsuya et al., 2016) and anxiety (Nomura et al., 2006) toward robots. Particularly relevant in the context of persuasive human-robot interaction is the psychological reactance which people experience when they sense their freedom in making decisions is at stake or constrained (Brehm, 1966; Rains and Turner, 2007). Psychological reactance can be limited to private thoughts such as reactant intentions, feelings of anger or negative thoughts toward the robot. However, it can also be manifested in physical expressions such as showing a dissatisfied face and through emotional communication such as shouting (Quick and Considine, 2008). Psychological reactance also can cause people not to comply, and even do something completely opposite from what they were asked to do because of strong persuasive attempts. Dillard and Shen (2005) proposed an intertwined model of reactance that consists of feelings of anger and negative cognitions for evaluating psychological reactance. Several experimental studies (Miller et al., 2006; Roubroeks et al., 2011; Ghazali et al., 2017) have used the intertwined model to measure human's psychological reactance when interacting with different social actors. Other than that, the strength (level of coercion) or intensity of language used by the actors (Roubroeks et al., 2011; Ghazali et al., 2017), goals in completing the given tasks (incongruent vs. congruent) (Roubroeks et al., 2010; Verberne et al., 2015) and social skill of the actors (Liu et al., 2008) can be sources of psychological reactance occurrence in persuasion activities.

As people respond to social cues (Atkinson et al., 2005; Lee et al., 2017) from technologies (Reeves and Nass, 1996), we anticipate that participants in our experiment will also show some social responses toward the social robot. Therefore, this paper reports an experiment that examines the influence of gender similarity between human and robot (similar vs. opposite genders) as well as the facial characteristics of the robot (least vs. most trustworthy). The social responses under study include users' trust in the robot and psychological reactance toward the interaction. Based on earlier research, we expect that gender similarity (Verberne et al., 2015) and the most trustworthy facial characteristics (Todorov and Oosterhof, 2011; Todorov et al., 2015) will evoke higher trust toward the robot. However, we cannot predict how similarity in gender and facial characteristics affects psychological reactance, which has not yet been examined by earlier research. Additionally, we predict that higher psychological reactance (caused by perceived loss of freedom) causes lower trust, as reported by Sue et al. (1998), Dowd et al. (2001), and Lee et al. (2014) in separate studies.

## Experiment design

This study examines the influence of gender similarity (similar vs. opposite) between a robot and a human upon the trust and psychological reactance the human feels toward the robot. It also examines whether facial characteristics engender trust in line with how Todorov et al. (2008) found that people judge trustworthiness from photos. Besides, this study also investigates how trust toward a robotic persuader can influence psychological reactance. Trust is measured in terms of trusting beliefs and trusting behaviors. Participants played a trust game inspired by the investment game (Berg et al., 1995; Xin et al., 2016) and the route planner game (de Vries, 2004), in which they were asked to make a drink for an alien.

**Figure 1:**
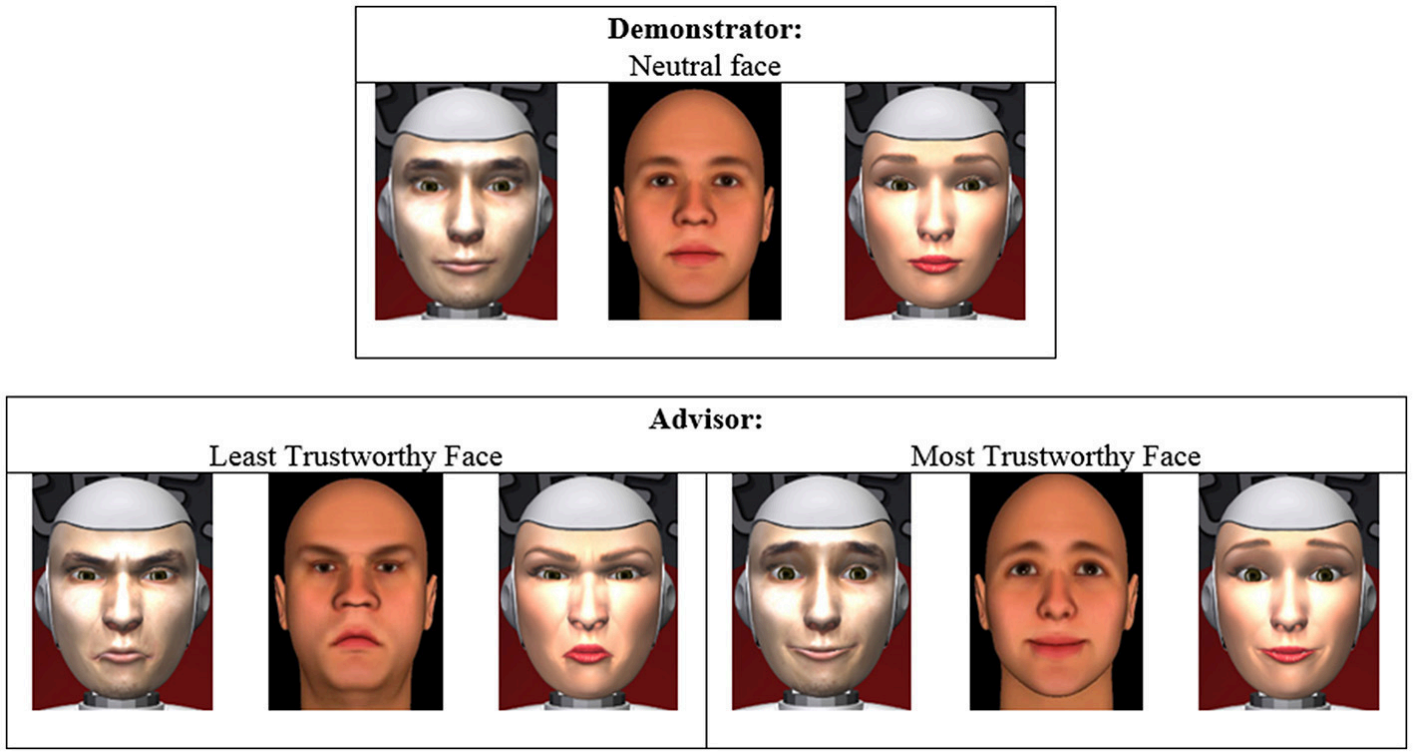
Facial characteristics of the demonstrator and the two advisors. For each case, there are corresponding: male robot **(Left)**, images from the study of Todorov and Oosterhof (2011) **(Center)**, and female robot **(Right)**.

More specifically, in this study, participants could decide between letting the robot choose the ingredients for the drink, thus exhibiting a trusting behavior (Vidotto et al., 2012), or selecting their own ingredients and thus demonstrating distrusting behavior toward the robot. Facial characteristics and gender were implemented in SociBot™, a robot featuring a human-like head on which an animated face is back-projected as shown in Figure 1. This apparatus allows displaying different facial characteristics, facial expressions, and human-like movements like blinking and lip synchronization.

The general task of the interaction is that the participant should create a beverage for an alien, which involves several choices for the ingredients. While making these choices, the robot serves as an advisor, assisting the participants in making their decision in selecting the ingredients for the beverage upon request. The hypotheses are presented in four parts, pertaining to trusting beliefs, trusting behaviors and psychological reactance:

**H1.Trusting beliefs**

H1(a). Participants will report higher trusting beliefs toward the robot with the most trustworthy face than the robot with the least trustworthy face
H1(b). Participants will report higher trust beliefs toward a robot of the same gender than toward a robot of the opposite gender

**H2.Trusting behaviors**

H2(a). Participants will request more help (trusting behaviors) from the robot with the most trustworthy face compared to the robot with the least trustworthy face
H2(b). Participants will request more help (trusting behaviors) from a robot with the same gender than from a robot with the opposite gender

**H3.Psychological reactance**

H3(a). Participants interacting with robot with the most trustworthy face will experience lower reactance compared to the one with the least trustworthy face
H3(b). Participants interacting with a robot of the same gender will experience higher reactance than participants interacting with a robot of the opposite gender

**H4. Correlation between psychological reactance and trust**

H4 Psychological reactance has a strong negative correlation to trust measures (trusting beliefs and trusting behaviors).

## Materials and methods

This study was carried out in accordance with the recommendations of Code of Ethics of the NIP (Nederlands Instituut voor Psychologen—Dutch Institute for Psychologists) and the research group on Human-Technology Interaction at Eindhoven University of Technology, with written informed consent from all subjects. All subjects gave written informed consent (in accordance with the Declaration of Helsinki). This study was reviewed and approved by the Human-Technology Interaction ethics board at Eindhoven University of Technology.

### Participants and design

Participants played a game with the SociBot™ which offered them persuasive advice and displayed different facial characteristics and gender according to the experimental condition. The experiment followed a 2x2 between-subjects design with facial characteristics (the most trustworthy face vs. the least trustworthy face) and gender similarity (similar vs. opposite) as independent variables. Seventy-two adult participants (41 males and 31 females) were recruited; with ages ranging between 18 and 47 (*M* = 23.90, *SD* = 4.15). Participants were given a reward for participation (€7.5 for university students and 9.5€ for external participants) and a different type of chocolate bar as a reward based on the participant's score during the game.

### Manipulations

#### Facial characteristics

During the experimental session, half of the participants played with the robot advisor that showed eyebrows pointing down and lips curled downwards at the edges: the least trustworthy facial characteristics according to Todorov et al. (2008, 2015). More specifically, in terms of the Facial Action Coding System (FACS) (Ekman and Friesen, 1978), facial characteristics that were altered (from the neutral face of the robotic advisor) were inner brow raiser, outer brow raiser, lips toward each other, upper lip raise, lip corner puller, dimpler, and lip pucker. The remaining participants played with SociBot™ featuring a face which was labeled as the trustworthy advisor with upturned eyebrows and lips. For the least trustworthy face, facial characteristics that differ from neutral face were: nasolabial deepener, lip corner depressor, lips toward each other, lip pucker, and lid tightener (Ekman and Friesen, 1978).

Both groups started the session by first interacting with the robot as a demonstrator. The demonstrator had the same gender as the participant and displayed neutral face and expression (refer to Figure 1 for more graphical details of the facial images) in order to establish a baseline context of the agent's facial characteristics and gender. Baseline conditions using neutral face were commonly used in earlier research (Kohler et al., 2009; Stuhrmann et al., 2011). This step was taken to allow controlling for individual differences in trusting somebody (in this case the robotic advisor). Thus, trusting beliefs and psychological reactance scores for the demonstrator were used as covariates in testing the hypotheses.

These facial characteristics (demonstrator and advisor's faces) were embedded into one robot only. The advisor (both with least trustworthy and most trustworthy faces) as well as the demonstrator resembled a human with light brown skin color tone and hazel eyes. English synthetic voice (produced with Acapela group software) was used to deliver the advice audibly originating from the robot's speaker. The robot also blinked its eyes at a natural rate. The experimenter controlled the sequence of advice by the robot from a different space using the Wizard of Oz technique. As the robotic agent consisting of a human-like head that connected with a torso via neck, SociBot™ was programmed to make human-like head postures (e.g., tilt/shakes) when delivering the advice.

#### Gender similarity

Two types of robot's gender were used in this study, which was the same (similar) or the opposite (opposite) gender of the advisor vs. the participant. The participants were asked to self-identify their gender as part of a demographic questionnaire, and the information given was 100% the same as the experimenter's observation. For participants in the similar gender condition, the advisor was given an identity as male (face and voice) if the participant identified himself as male while a female advisor was used if the participant is female. In contrast, the advisor's gender would be opposite to the participant's gender in the opposite gender condition. The gender for the demonstrator was always the same gender as the participant.

### Task

The experimental task was to play the “Beverages Creation Station” game, a hybrid between an online game called “Smoothie Maker: Creation Station” (URL: https://www.youtube.com/watch?v=6Rh3BATmps0) and a trust game (Berg et al., 1995; de Vries, 2004). In the original game, players have 100% freedom to make their own favorite smoothie by making a series of decisions, of which none can be right or wrong. We made several adaptations in our developed game. First, we emphasized that among all selections given for each task, there was only one right answer, thus stimulating participants to think before choosing. Second, the number of tasks was increased to 10 compared to only four in the original game. Another six tasks were added in order to extend the duration of the session and thus allow trust toward the agent to grow. We also added a “score” to the graphical user interface of the game and an option for asking the agent to suggest a choice.

The trust game was implemented as follows: Each participants was given 20 credits at the start of the game. Every move costs one credit, but if the participant asks for help from the advisor, it costs 2 credits. Participants win 4 credits for every correct choice they make. Participants are only informed what the right choices were after the end of the game.

The social agent used direct and high controlling language based on the study of Ghazali et al. (2017) who found that forceful language in persuasion activity by robot leads to higher compliance and lower reactance, e.g., “*You are obliged to pick the third design”* and “*Definitely, choose honey!”*

### Procedure

**Figure 2:**
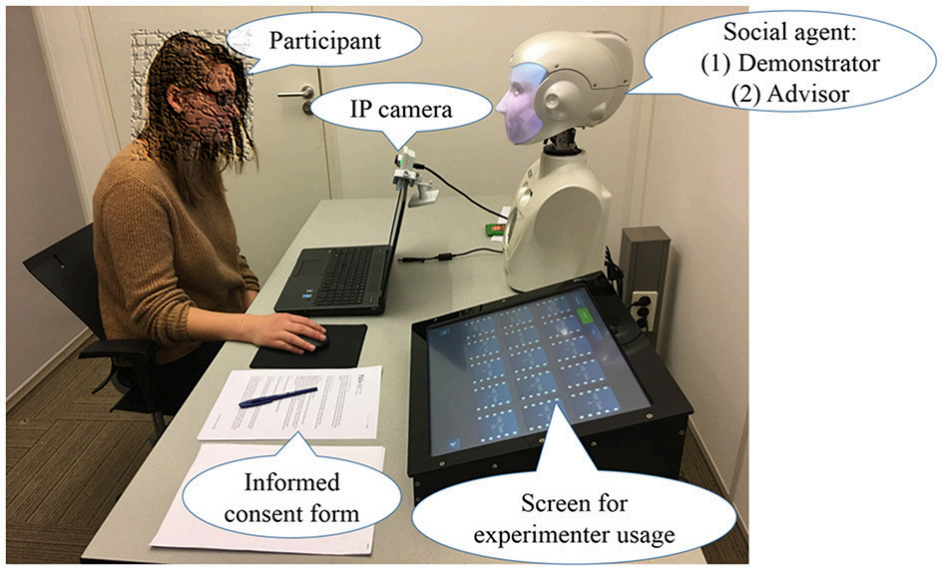
Experimental setup. A written informed consent had been obtained from the individual for the publication of this image.

Participants sat on a chair facing the robot. A laptop that was placed in front of the participants was used to fill in questionnaires and play the game (see Figure 2). An IP camera attached to the laptop screen recorded participants' facial expression while playing the game. The experiment consists of three phases: (1) Introduction [5 min] (2) Demonstration [10 min] (3) Experiment [30 min].

In the first phase, participants gave informed consent and demographic information, and the experimenter summarized the experimental procedure.

In the second phase, the experimenter introduced the first robotic character called the “demonstrator” and demonstrated how to play the game. Before the experimenter would leave the room, the participant was reminded that the robot was on the same team as the participant and had the responsibility to help the participant achieve the highest score possible. The experimenter also reminded the participant that it is up to them whether to trust the advisor in the selection process. Then, participant could fill in a questionnaire consisting of evaluative questions regarding their impression of the demonstrator.

In the third phase, participant played the game. During this phase, the robot would assume the character of the advisor. The advisor greeted the participant by introducing itself as “Hello, I am your advisor” to make participant aware of the changed role of the agent. After making all selection tasks, the second questionnaire appeared on the screen as a Google form labeled as the “Advisor Questionnaire” in which, the participant were asked to evaluate their experience of playing the game together with the advisor.

### Measure

#### Trusting beliefs

We measured trusting beliefs with a questionnaire using the scale developed by Jian et al. (2000), perceived trust by using a scale by Tay et al. (2014) and a scale by Heerink et al. (2009), as well as individualized trust evaluations by using a scale developed by Wheeless and Grotz (1977). We combined the overlapping questions as appropriate. For example, both trust scale items (Jian et al., 2000) and the individual trust scale (Wheeless and Grotz, 1977) ask how much a participant thinks the advisor is honest. The combined trusting beliefs questionnaire includes two sets of items:

- The Likert scale of 7 levels ranging from completely disagree to completely agree toward the following statements: (from trust scale items questionnaire (Jian et al., 2000) *The advisor behaves in an ethical manner, I am confident of the intentions, actions, and outputs of advisor, I am not wary of the advisor, I am confident with the advisor*. Another three Likert scales of 7 levels inquired agreement with statements that were adapted from the perceived trust questionnaires (Heerink et al., 2009; Tay et al., 2014) including: *I will trust the advisor if the advisor gives me advice again in the future, I trust that the advisor can provide me correct answers to the game*, and *I will follow the advice that the advisor gives me*.
- Nine semantic differential items with seven levels adapted from the individualized trust scale questionnaire (Wheeless and Grotz, 1977) with the following poles: *untrustworthy-trustworthy, unreliable-reliable, insincere-sincere, dishonest-honest, distrustful-trustful, inconsiderate-considerate, divulging-confidential, deceitful-not deceitful*, and *disrespectful-respectful*.

A reliability analysis showed that the various components of our combined trusting beliefs questionnaire were highly correlated. By combining all the questionnaire items (described above), we were able to construct a highly reliable (Cronbach's α = 0.96; 16 items) trusting beliefs measure.

#### Trusting behaviors

The game allows a clear behavioral measure of trust, namely how many times participants ask the help of the advisor. For example, if a particular participant would ask help from the designated advisor only for tasks 1, 5, 6, and 8 while answering the remaining six tasks independently, then he/she would be given the trusting behaviors score of 4.

#### Psychological reactance

Based on the intertwined model of reactance by Rains and Turner (2007) as well as Dillard and Shen (2005), we took two measures of psychological reactance based on self-report: feelings of anger and negative cognitions. Feelings of anger were rated on a 5-point Likert Scale measuring participants' level of *irritation, angriness, annoyance*, and *aggravation* toward the advisor.

To measure negative cognitions, we asked participants to write down the thoughts they had in their mind after playing the game with the advisor and label each of them as negative (N), positive (P), or neutral (Neu). As we were not interested in positive and neutral cognitions, only the negative cognitions score was calculated using the same steps highlighted by Dillard and Shen (2005). This step involves separation of negative cognitions (used as negative cognitions' score) and negative emotions (that was taken out from the negative cognitions score) (Shaver et al., 1987).

Finally, the facial emotional expression of the participants while interacting with the advisor were captured and analyzed using a software called FaceReader (Barakova et al., 2015; Adams et al., 2016) which is based on Facial Action Coding System (FACS) (Ekman and Friesen, 1978). We counted the instances where FaceReader would classify a facial expression as angry to obtain a behavioral measure of reactance.

A reliability analysis on the proposed psychological reactance elements: feelings of anger, negative cognitions, and facial emotion (angriness as detected by the FaceReader software) showed that the Cronbach's α increased by eliminating the measurement based on the facial expression of emotion. Therefore, we constructed a reliable (Cronbach's α = 0.89) measure of psychological reactance by averaging a user's scores on feelings of anger and negative cognitions only.

#### Exploratory measures

A number of extra measures were taken to support exploratory analysis: a semantic differential scale with endpoints masculine/feminine, and 7-point scales to rate the following properties: healthy, and attractive (Verberne et al., 2015).

To measure how much participants liked the designated advisor, we used the partner liking rate scale by Guadagno and Cialdini (2002) which includes thirteen 7-point Likert scales assessing partners by the following characteristics: *approachable, confident, likeable, trustworthy, interesting, friendly, sincere, warm, competent, informed, credible, modest*, and *honest*.

The degree of anthropomorphism and perceived intelligence of the advisor were rated using 5-point semantic differentials from the Goodspeed Questionnaire (Bartneck et al., 2009) indicating that “*The advisor was*”: *fake/natural, machinelike/human-like, unconscious/conscious, artificial/lifelike*, and *moving rigidly/moving elegantly* for anthropomorphism factor; whereas *incompetent/competent, ignorant/knowledgeable, irresponsible/responsible, unintelligent/intelligent*, and *foolish/sensible* for perceived intelligence.

## Results

All statistical analyses carried out in this study used Statistical Package for Social Science (SPSS) version 23. Results are presented in three parts concerning respectively, manipulation checks, hypothesis testing, and exploratory analysis.

### Manipulation check

An examination of participant's perception of advisor's gender reveals the main effect of our masculinity/ feminine manipulation, *F*_(1, 70)_ = 1317.8, *p* < 0.001 using one-way Analysis of Variance (ANOVA) test. A Brown-Forsythe test of equality of means revealed a significant relationship between the perception of advisor's gender (feminine vs. masculine) and the advisor's gender, *F*_(1, 64.71)_ = 1601.86, *p* < 0.001, which means that the gender of the robot was perceived correctly by all participants. In addition, the female advisor was perceived as more feminine (*M* = 6.07, *SD* = 0.83, *n* = 30) than the male advisor perceived as masculine (*M* = 5.50 *SD* = 1.60, *n* = 42).

### Hypothesis testing

We conducted statistical analyses in testing the hypotheses after ensuring that the conditions and assumptions for the tests (e.g., ANCOVA etc.) were met.

#### Trust

This study intended to combine trusting beliefs and trusting behaviors components into one measurement that we named trust. A Pearson correlation test was run to check if there is a correlation between these two measurements and also to check the strength of the correlation. As a result, it was found that there was no significant (*n.s*) correlation between the two components, *r* = 0.15, *p* = 0.20. Based on this outcome, the hypothesis for trust was split into two different hypotheses: trusting beliefs and trusting behaviors.

##### Hypothesis 1: trusting beliefs

*Hypothesis 1(a)*. The result from Univariate Analysis of Covariance (ANCOVA) is consistent with *H1(a)* which predicts that the advisor with the most trustworthy facial characteristics (i.e., with eyebrows pointing down and lips curled at the edges) would attain higher trusting beliefs than the least trustworthy face's advisor. By using the trusting beliefs score on demonstrator as a covariate, a significant difference was found [*F*_(1, 69)_ = 16.61, *p* < 0.001, partial η^2^ = 0.19] between the trustworthiness of the advisor with the most trustworthy facial characteristics (*M* = 5.29, *SD* = 0.88) and the advisor with the least trustworthy facial characteristics (*M* = 4.38, *SD* = 1.04).

*Hypothesis 1(b)*. An ANCOVA found no main effect of gender similarity (between the advisor and participants) on trusting beliefs, *F*_(1, 69)_ = 0.001, *p* = 0.98 (*n.s*.) for which hypothesis *H1(b)* is rejected. Thus, having similar or opposite gender does not lead to reporting different trusting beliefs (see Table 1).

**Table 1:** Mean scores on trusting beliefs (and standard deviations between brackets) for the gender similarity manipulation.

**Advisor's gender**	**Participants' gender**	***Mean* (*SD*)**	***N***
Male	Male	5.06 (0.58)	23
	Female	4.68 (1.24)	18
Female	Male	4.97 (1.26)	18
	Female	4.43 (1.10)	13

When disentangling gender similarity into its components of participants' gender and advisor's gender, results (as shown in Table 1) show that male participants reported slightly higher trusting beliefs toward the advisor (*M* = 5.02, *SD* = 0.92) (regardless of the advisor's gender or the facial characteristics of the advisor) as compared to the female participants (*M* = 4.57, *SD* = 1.17). Presenting evidence for this difference, an ANCOVA using facial characteristics and participants' gender were as independent variables, the trusting beliefs score toward the advisor as the dependent variable, and postulated the trusting beliefs score toward the demonstrator as a covariate, showed a main effect of participants' gender, *F*_(1, 67)_ = 6.38, *p* = 0.01, partial η^2^ = 0.09. Gender of the advisor had no independent effect, *F* < 1, nor did analyses show interactions between participants' gender or advisors' gender and the facial characteristics of the advisor, all *F*'s < 1.

**Figure 3:**
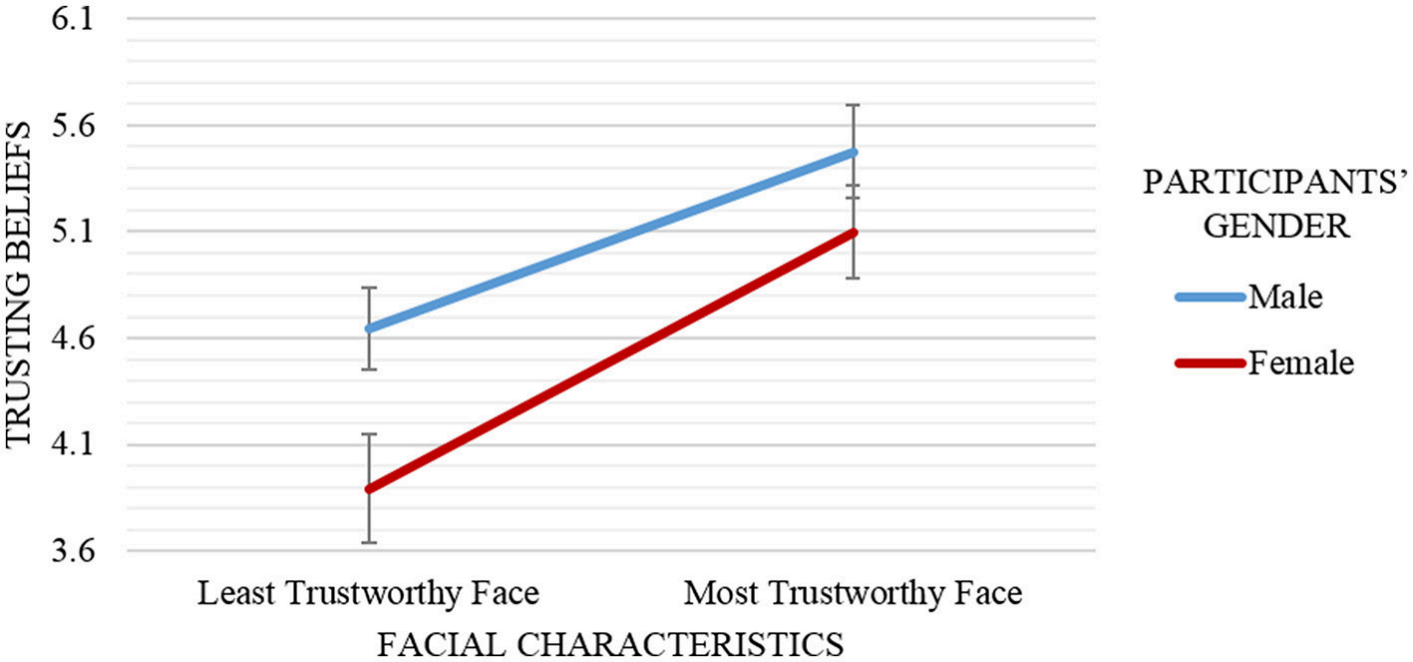
Mean and standard error of trusting beliefs scores by advisor's facial characteristics (least trustworthy face vs. most trustworthy face) and participants' gender (male vs. female). Male participants reported higher trusting beliefs about an advisor (independent of the advisor's facial characteristics) compared to female participants. Participants (independent of their gender) reported higher trusting beliefs about the most trustworthy face advisor (vs. the least trustworthy face advisor). Results showed no interaction effect between participants' gender and the advisor's facial characteristics on trusting beliefs.

As Figure 3 depicts, female participants held the lowest trusting beliefs toward the advisor with the least trustworthy face (*M* = 3.89, *SD* = 1.23), while male participants rated the advisor with the most trustworthy face as the most trustworthy advisor (*M* = 5.48, *SD* = 0.87).

Several conclusions stem from these analyses. First, the trusting beliefs toward the least trustworthy face were always lower than toward the most trustworthy face regardless of the participants' gender [in line with *H1(a)*]. Second, male participants were successfully persuaded to believe that the advisor was more trustworthy than female participants (adjacent to the outcome in *H1(b)*). Overall, female participants held the lowest trusting beliefs toward the advisor with the least trustworthy face while male participants rated the advisor with the most trustworthy face as the most trustworthy advisor.

In summary, these analyses demonstrate clearly that robots with facial characteristics that humans consider trustworthy enhance trusting beliefs toward the robot, regardless of its gender. Moreover, this effect seems to be stronger for male participants rather than female participants.

##### Hypothesis 2: trusting behaviors

*Hypothesis 2(a)*. Analysis of the trusting behaviors score revealed a main effect of the facial characteristics manipulation, *F*_(1, 70)_ = 4.12, *p* = 0.05, partial η^2^ = 0.06 using one-way ANOVA test. On average, participants showed higher trusting behaviors toward an advisor with the most trustworthy face, *M* = 5.31, *SD* = 2.41 than toward an advisor with the least trustworthy face, *M* = 4.25, *SD* = 1.98. Overall, almost in all tasks, participants who interacted with the most trustworthy face's advisor preferred to ask the advisor to solve the tasks given on behalf of them than the least trustworthy face's advisor.

**Figure 4:**
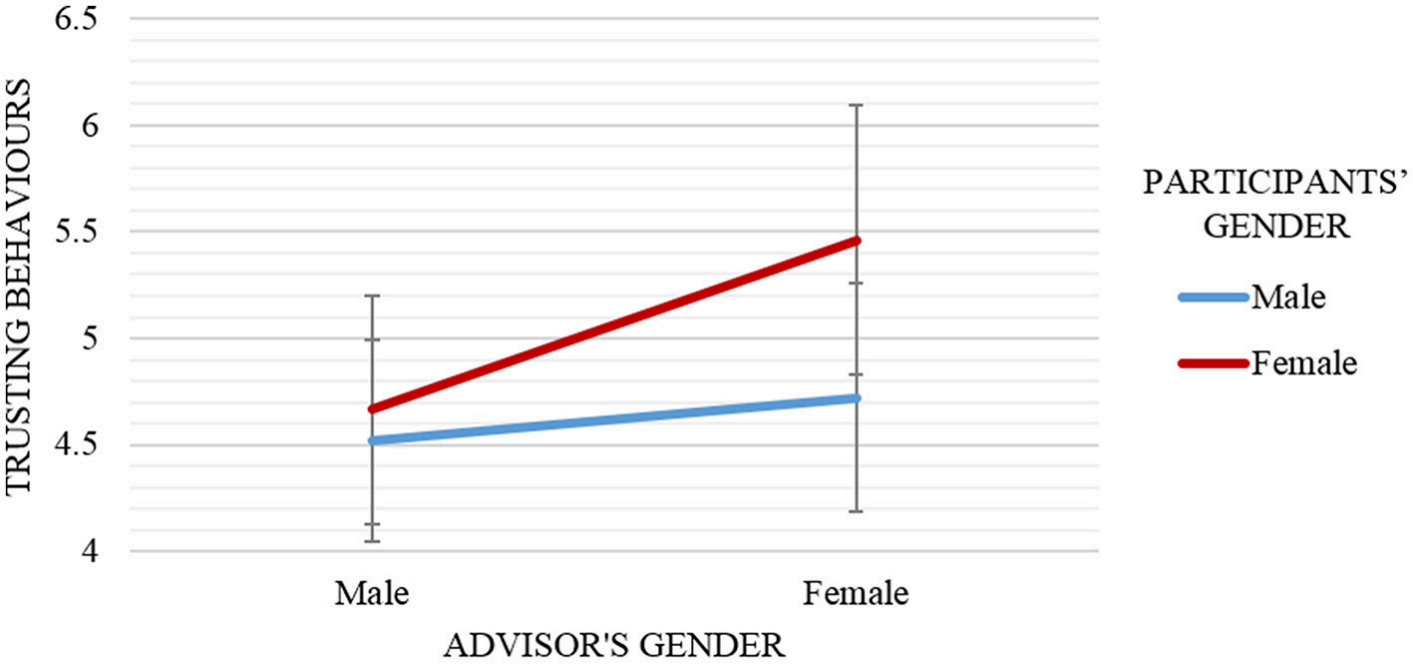
Mean and standard error of trusting behaviors scores by advisor's gender (male vs. female) and participants' gender (male vs. female). Participants (independent of their gender) reported higher trusting behaviors about the female advisor (vs. male advisor). Female participants reported higher trusting behaviors about an advisor (independent of the advisor's gender) than male participants.

*Hypothesis 2(b)*. To test whether gender similarity increased trusting behaviors, two separate Multivariate Analysis of Variance (MANOVA) analyses were run and the results found (a) no significant main effects of gender similarity, *F*_(1, 70)_ = 0.10, *p* = 0.76, partial η^2^ = 0.001, and (b) no interaction effect of the manipulations of participants' gender and advisor's gender on trusting behaviors score, *F*_(1, 68)_ = 0.29, *p* = 0.59, partial η^2^ = 0.004 (see Figure 4).

Figure 4 suggests that the female advisor induced higher trusting behaviors (*M* = 5.03, *SD* = 2.50) than the male advisor (*M* = 4.59, *SD* = 2.06) regardless of the participants' gender. Further statistical exploration was done to investigate whether either male or female participants tend to show higher trusting behaviors toward the advisor (by neglecting the advisor's gender). It can be seen from the graph in Figure 4 that female participants (*M* = 5.00, *SD* = 2.45) reported higher perceived trusting behaviors than male participants (*M* = 4.61, *SD* = 2.11).

In summary, these results suggest that people show more trusting behaviors toward a robot with facial characteristics they trust on humans, which does not seem to be affected gender similarity, while there is some experimental evidence that they appear to show higher trusting behaviors to a female robot more than a male robot.

#### Psychological reactance

##### Hypothesis 3

*Hypothesis 3(a)*. To test whether facial characteristics influence psychological reactance, we conducted a repeated measures Analysis of Covariance (ANCOVA) after ensuring that all conditions and assumptions for this test were met (e.g., we found no evidence for multicollinearity, extreme outliers, or non-normal distribution).

**Figure 5:**
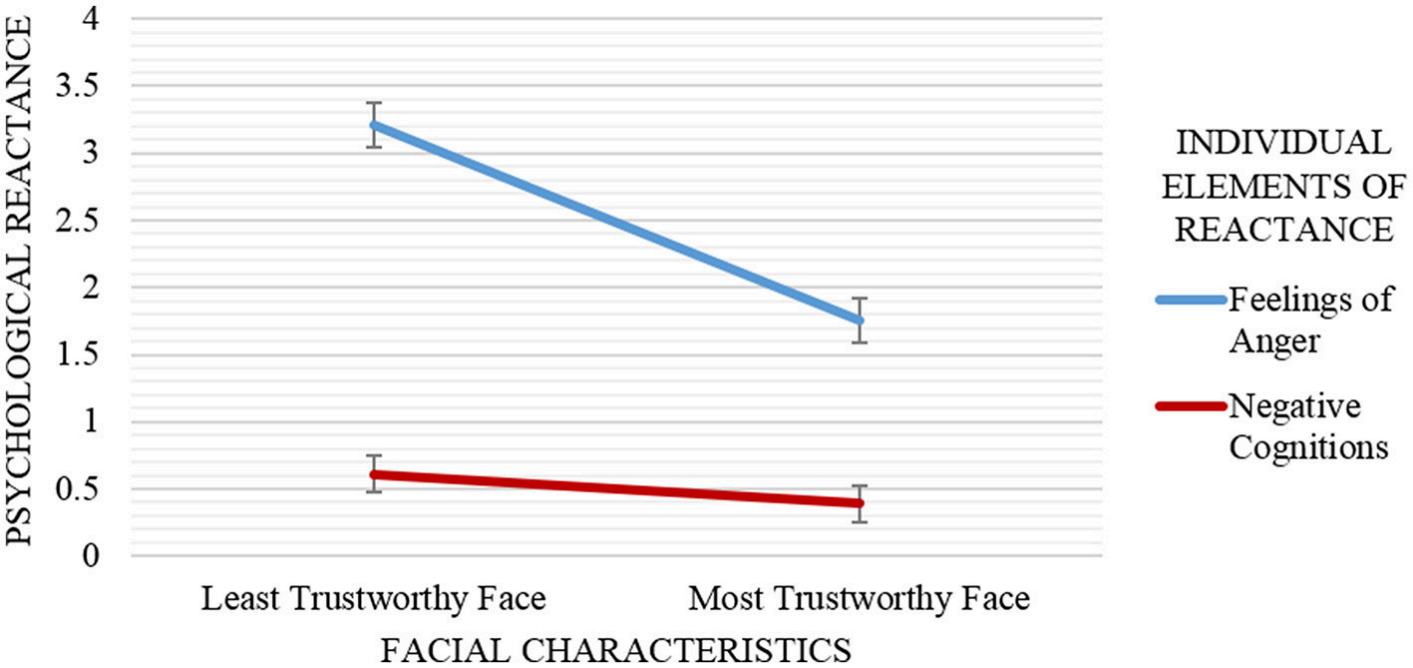
Mean and standard error of psychological reactance elements (feelings of anger and negative cognitions) scores by facial characteristics (least trustworthy face vs. most trustworthy face). Participants reported lower psychological reactance (and significantly lower feelings of anger) when interacting with the most trustworthy face advisor than the least trustworthy face advisor. Results showed no significant main effect of facial characteristics on negative cognitions.

Facial characteristics were used as the independent variables, psychological reactance (measured by feelings of anger and negative cognitions) as the dependent variable, and psychological reactance evaluations in the demonstration session (feelings of anger and negative cognitions toward demonstrator) as the covariate. The result demonstrates a significant main effect of facial characteristics on psychological reactance, *F*_(1, 68)_ = 22.94, *p* < 0.001. The lowest psychological reactance recorded by the participants in the most trustworthy face condition (*M* = 1.07, *SD* = 0.72) and the highest reactance experienced by the participants who interacted with the least trustworthy faced advisor (*M* = 1.91, *SD* = 0.72) (see Figure 5).

The facial characteristics manipulation resulted a significant main effect for feelings of anger toward the advisor (with feelings of anger toward demonstrator as a covariate), *F*_(1, 69)_ = 38.25, *p* < 0.001, partial η^2^ = 0.36. However, no significant main influence of facial characteristics was found on negative cognitions score (with negative cognitions toward demonstrator as a covariate), *F*_(1, 69)_ = 1.34, *p* = 0.25, partial η^2^ = 0.02. The mean difference of feelings of anger score for the least trustworthy face advisor and the most trustworthy face advisor was 1.46 points (with a 95% confidence interval [0.99, 1.93]) higher for the least trustworthy face advisor than for the most trustworthy face advisor.

*Hypothesis 3(b)*. The second hypothesis for psychological reactance predicted that participants who interacted with an advisor of a similar gender would experience higher psychological reactance compared to the participants in the opposite gender condition. To test this hypothesis, the psychological reactance score for the advisor (feelings of anger and negative cognitions) was submitted to gender similarity (similar vs. opposite) x 2 (repeated measure of feelings of anger and negative cognitions toward advisor) x 2 (repeated measure of feelings of anger and negative cognitions for demonstrator as covariates) in ANCOVA test. There was no significant main effect of gender similarity on psychological reactance, *F*_(1, 68)_ = 0.07, *p* = 0.80. So, overall, not supporting our hypothesis, results provided no evidence that the participants who interacted with similar gender's advisor (*M* = 1.47, *SD* = 0.14) experienced lower or higher psychological reactance than the participants who interacted with opposite gender's advisor (*M* = 1.52, *SD* = 0.14). However, results also showed that the effect of gender similarity on psychological reactance was different for the two components of psychological reactance, indicated by an interaction of gender similarity x psychological reactance component (repeated measure of feelings of anger and negative cognitions toward advisor), *F*_(1, 68)_ = 4.70, *p* = 0.08. Further explorations of the relationship between the dependent and independent variables in verifying this hypothesis are elaborated in Table 2 by separating the psychological reactance component into individual measures of feelings of anger and negative cognitions.

**Table 2:** Mean scores on psychological reactance elements (and standard deviations between brackets) for the gender similarity manipulation.

**Gender similarity**	**Psychological reactance**	
	**Feelings of anger**	**Negative cognitions**
Similar	2.64 (1.07)	0.31 (0.52)
Opposite	2.33 (1.38)	0.69 (1.01)

Simple effect analyses show that there was no statistical significant difference of gender similarity on feelings of anger, *F*_(1, 69)_ = 0.96, *p* = 0.33, partial η^2^ = 0.01. Still, the influence of gender similarity was significant on negative cognitions, *F*_(1, 69)_ = 4.10, *p* = 0.05, partial η^2^ = 0.06. That is, results provided no evidence that when participants interacted with the advisor that has similar gender to them, the feelings of anger (*M* = 2.64, *SD* = 1.08) were higher or lower compared to the participants in opposite gender interactions (*M* = 2.33, *SD* = 1.38). In contrast, the negative cognitions for the participants in the similar gender conditions (*M* = 0.31, *SD* = 0.52) was lower than the participants in opposite gender conditions (*M* = 0.69, *SD* = 1.01).

**Figure 6:**
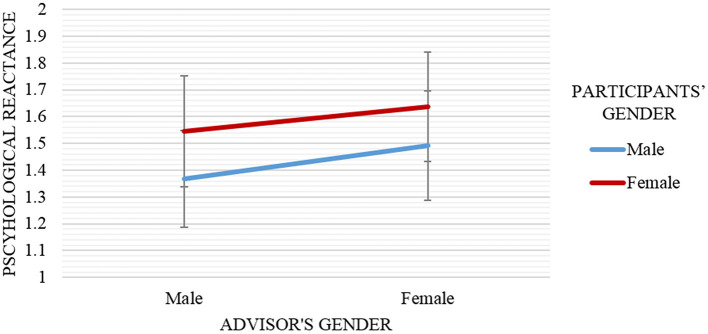
Mean and standard error of psychological reactance scores by advisor's gender (male vs. female) and participants' gender (male vs. female). Overall, participants that interacted with similar gender advisor (e.g., male participants paired with male advisor) reported lower psychological reactance, especially negative cognitions compared to opposite gender advisor (e.g., male participants paired with female advisor).

A repeated measure ANCOVA test was run with participants' and advisor's genders as independent variables, psychological reactance toward advisor as a dependent variable, and psychological reactance toward demonstrator as a covariate. Result revealed no significant interaction effect between those variables, *F*_(1, 66)_ = 0.01, *p* = 0.94, partial η^2^ = 0.07 as demonstrated in Figure 6.

Figure 6 shows that male participants (*M* = 1.43, *SD* = 0.82) always recorded the lowest psychological reactance compared to female participants (*M* = 1.57, *SD* = 0.87) in regards to the advisor's gender. Besides, it can also be concluded that female advisor (*M* = 1.55, *SD* = 0.83) provoked higher psychological reactance to occur during the interaction compared to male advisor (*M* = 1.44, *SD* = 0.85). More importantly, male participants experienced higher psychological reactance when interacted with the opposite gender advisor. That is, female advisor (*M* = 1.49, *SD* = 0.99) and male advisor (*M* = 1.38, *SD* = 0.69). However, the psychological reactance score for female participants was lower when they were interacted with opposite gender advisor. That is, male advisor (*M* = 1.53, *SD* = 1.04) and female advisor (*M* = 1.63, *SD* = 0.58).

In summary, the main finding from this analysis is that psychological reactance (especially negative cognitions) is lower when the robot has a similar gender to the human persuadee. Further, psychological reactance (especially feelings of anger) is lower when the robot featured trustworthy facial characteristics.

#### Correlation between trust and psychological reactance

##### Hypothesis 4

A Pearson product-moment correlation coefficient was computed to assess the relationship between trusting beliefs, trusting behaviors, and psychological reactance (dependent variables) that were used in the previous hypotheses. No significant correlation was found between psychological reactance and trusting behaviors *r* = −0.02, *p* = 0.85 *(n.s.)*. A strong negative correlation (2-tailed) was found between trusting beliefs and psychological reactance, *r* = −0.74, *p* < 0.001. Thus, a drop in psychological reactance was correlated with higher trusting beliefs, but not with trusting behaviors.

### Exploratory analysis

#### Inherent confounds on facial characteristics manipulation

**Figure 7:**
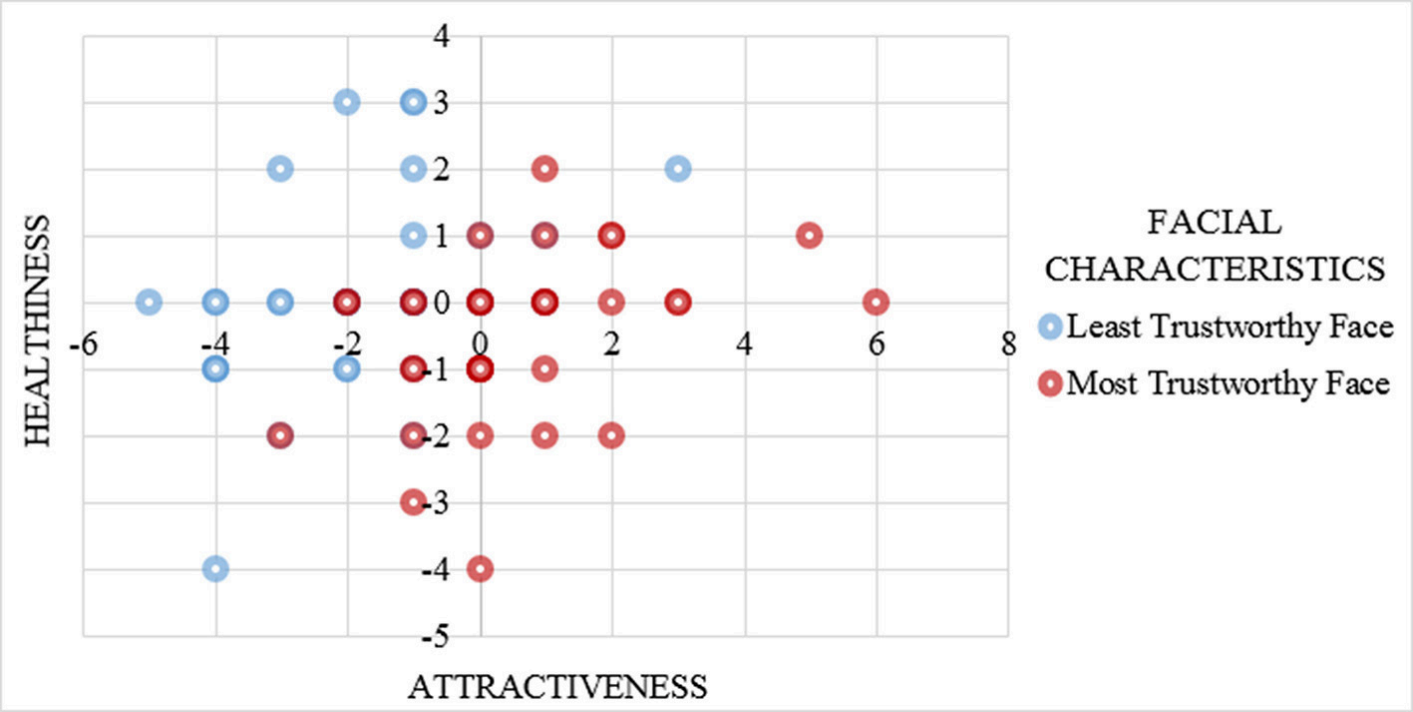
Mean attractiveness and healthiness of the advisor scores by advisor's facial characteristics (least trustworthy face vs. most trustworthy face) with neutral facial characteristics as 0. Participants reported higher attractiveness and healthiness scores about the most trustworthy face advisor (vs. least trustworthy face advisor).

To assess the manipulation involving the facial characteristics of the advisor, a MANOVA test was performed using attractiveness and healthiness scores as dependent variables, the least and the most trustworthy faces of the advisor as the independent variable. The demonstrator with the neutral face was used as a baseline for these measurements (attractiveness and healthiness of neutral facial characteristics are equal to zero) and the difference of scores of attractiveness and healthiness between the demonstrator and the advisor were examined. Results shows a significant main effect of facial characteristics on the robot's attractiveness and healthiness scores with *Wilks'* Λ = 0.71, *F*_(2, 69)_ = 14.16, *p* < 0.001. Figure 7 shows the scatter plot of the advisor's facial characteristics vs. attractiveness and healthiness scores of the agent.

For the attractiveness factor, an advisor with the most trustworthy face scored slightly higher than neutral attractiveness score by *M* = 0.53 (*SD* = 1.72) while an advisor with the least trustworthy face fall in the unattractive range, *M* = −1.69, *SD* = 1.72. An ANOVA test confirmed a significant main effect of facial characteristics of the advisor on the attractiveness, *F*_(1, 70)_ = 28.46, *p* < 0.001, partial η^2^ = 0.29. Moreover, result show marginal significant main effect of the healthiness measure with facial characteristics, *F*_(1, 70)_ = 3.74, *p* = 0.057, partial η^2^ = 0.05. Descriptive statistics for this test revealed that the advisor with the least trustworthy face was less healthy (*M* = −0.53, *SD* = 1.52) compared to the advisor with the most trustworthy face (*M* = 0.08, *SD* = 1.13).

#### Mediation analysis

**Figure 8:**
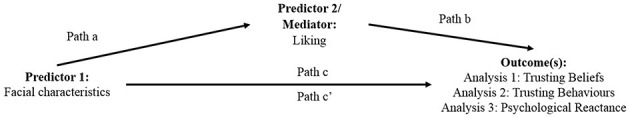
Liking rate fully mediates the relationship between facial characteristics and both trusting beliefs (in analysis 1) and psychological reactance (in analysis 3). However, liking rate did not mediate the relationship between facial characteristics and trusting behaviors (in analysis 2).

To test suspected mediation between the dependent and independent variables, three mediation analyses (one for each dependent variable stated in the hypothesis: trusting beliefs, trusting behaviors, and psychological reactance) were conducted following the steps of mediation analysis developed by Baron and Kenny (1986). Model testing hypotheses for mediation analysis 1, mediation analysis 2 and mediation analysis 3 can be seen in Figure 8. Details for each mediation analysis was described in the following subsections.

##### Trusting beliefs

Regression analysis was used to investigate whether liking mediates the effect of facial characteristics on trusting beliefs. First, this analysis showed that facial characteristics were a significant predictor of trusting beliefs (*B* = 0.43, *SD* = 1.95), *t* = 3.96, *F*_(1, 70)_ = 15.68 (path c). Second, we checked for a positive relationship between facial characteristics and liking. Results confirmed that facial characteristic was a significant predictor of liking (*B* = 0.56, *SD* = 1.95), *t* = 5.71, *F*_(1, 70)_ = 32.63 (path a). Third, we checked whether the mediator (liking) affected the outcome (trusting beliefs). Indeed, liking was a significant predictor of trusting beliefs score (*B* = 0.89, *SD* = 0.42), *t* = 15.97, *F*_(1, 70)_ = 255.02 (path b). Finally, this analysis showed that the effect of facial characteristics on trusting beliefs became non-significant when taking into account liking a mediator (*B* = −0.11, *SD* = 1.19), *t* = −1.58, *F*_(2, 69)_ = 131.49 (path c').

These results support the hypothesis that liking is a full mediator of the relationship between facial characteristic and trusting beliefs.

##### Trusting behaviors

To investigate whether liking mediates the effect of facial characteristics on trusting behavior, we conducted a second linear regression analysis. First, this analysis showed that facial characteristics were a significant predictor of trusting behavior (*B* = 0.24, *SD* = 4.41), *t* = 2.03, *F*_(1, 70)_ = 4.12 (path c). Next, results confirmed that facial characteristics was a significant predictor of liking (*B* = 0.56, *SD* = 1.95), *t* = 5.71, *F*_(1, 70)_ = 32.63 (path a). Again, we checked whether the mediator (liking) affected the outcome (trusting behavior). Indeed, liking was not a significant predictor of trusting behavior (*B* = 0.11, *SD* = 1.95), *t* = 0.91, *F*_(1, 70)_ = 0.83 (path b). As the relationship in path b was not significant, it can be concluded that mediation was not possible.

Thereby, we reject the hypothesis that liking is a mediator of the relationship between facial characteristic and trusting behaviors.

##### Psychological reactance

The third linear regression analysis was conducted to investigate whether liking mediates the effect of facial characteristics (causal variable) on psychological reactance. Because (as described above) results showed that facial characteristics influenced (as the main effect) the repeated measure (combining measure negative cognitions and feelings of anger) of psychological reactance, we calculated a psychological reactance score for each participant by averaging the participant's score on feelings of anger and on negative cognitions. First, this analysis showed that facial characteristics were a significant predictor of psychological reactance (*B* = −0.50, *SD* = 1.44), *t* = −4.86, *F*_(1, 70)_ = 23.64 (path c). Next, results confirmed that facial characteristics was also a significant predictor of liking (*B* = 0.56, *SD* = 1.95), *t* = 5.71, *F*_(1, 70)_ = 32.62 (path a). Again, we checked whether the mediator (liking) affected the outcome (psychological reactance). Indeed, liking was a significant predictor of psychological reactance (*B* = −0.81, *SD* = 0.42), *t* = −11.46, *F*_(1, 70)_ = 131.37 (path b). Finally (step 4), this analysis showed that the effect of facial characteristics on psychological reactance became non-significant when taking into account liking as a mediator (*B* = −0.07, *SD* = 1.19), *t* = −0.81, *F*_(2, 69)_ = 65.69 (path c').

These results support the hypothesis that liking is a full mediator of the relationship between the facial characteristic and psychological reactance.

#### Anthropomorphism and perceived intelligence

A two-way ANOVA was conducted to determine if the anthropomorphism score of the social agent (in this case the advisor) was biased by the manipulations of facial characteristics portrayed by the advisor and gender similarity of the advisor and participants. There was no significant relationship found between all three measured factors, namely, facial characteristics and gender similarity (independent variables) toward anthropomorphism (dependent variable), *F*_(1, 68)_ = 1.26, *p* = 0.27 *(n.s)*. Thus, the independent variables used in this study did not increase the anthropomorphism value of the agent used (as an advisor) during the interaction.

Furthermore, an ANOVA test showed that there was no significant effect of facial characteristics and gender similarity of the advisor and participants upon perceived intelligence, *F*_(1, 68)_ = 1.55, *p* = 0.22 *(n.s.)* but there was a significant relationship between facial characteristics on the perceived intelligence with *F*_(1, 68)_ = 5.48, *p* = 0.02, partial η^2^ = 0.07. Additional analysis on the influence of facial characteristics toward perceived intelligence revealed that the most trustworthy face (*M* = 3.84, *SD* = 0.60) was perceived as more intelligent compared to the least trustworthy face (*M* = 3.49, *SD* = 0.69).

## Discussion

In this study, participants were asked to play a trust game with a social robot, where the social robot attempted to persuade the participants regarding 10 different choices for making a beverage for an alien. The advisor's facial characteristics (least trustworthy face vs. most trustworthy face) and gender similarity (similar vs. opposite) were manipulated in a between-subjects experiment. Participants' trusting beliefs, trusting behavior, and psychological reactance responses were measured. In line with theories like the media equation hypothesis (Reeves and Nass, 1996), it was expected that basic social characteristics suffice to elicit social responses. So, we anticipated that participants in our experiment would also show some social responses (trust and psychological reactance) toward the social robot.

As expected (H1), the advisor's facial characteristics had a significant effect on the trusting beliefs toward the advisor. That is, participants reported higher trusting beliefs toward the advisor with the most trustworthy facial characteristics than toward the one with the least trustworthiness face. Regardless of the advisor's gender, male participants (compared to female participants) believed that the robotic advisor could be trusted more. Thus, it can be suggested that facial characteristics (the most trustworthy face's advisor) are essential for persuading the participants (especially male participants) to evaluate the robotic advisor to be trustworthy. This result is in line with neuropsychological research (Todorov et al., 2008), which suggested that the response to trustworthy faces is hard-coded in our brains; there is a part of the human brain (the amygdala) that responds to trust-related facial characteristics of faces presented on-screen. Although the current research did not investigate brain area activation, our study showed that facial characteristics of the robot activated trustworthiness judgments, just as was found in these earlier studies (Jeanquart-Barone and Sekaran, 1994; Todorov and Oosterhof, 2011). Furthermore, the current research extends earlier research in social psychology (Jeanquart-Barone and Sekaran, 1994; Todorov and Oosterhof, 2011) showing that facial characteristics of a distinctly non-human, robotic social entity can activate trustworthiness judgments and trusting behavior. Earlier research (Todorov et al., 2008, 2015) showed that facial characteristics of artificial faces on the screen could influence trustworthiness judgments of human perceivers. Importantly, the current results are the first to show these effects in the context of human-robot interaction.

Our results did not find the expected influence of gender similarity on trusting beliefs (H1(b)). That is, participants did not report significantly more trusting beliefs for the robot having the same gender as them. This finding did not confirm earlier studies that suggested that gender similarity (Byrne, 1997) between the participants (users) and the advisor (robotic partner) influence trust (Goetz et al., 2003; Eyssel et al., 2012). A potential explanation might be that the advisor's task was not associated with any explicit gender stereotypes, so participants held no expectations as to whether a male or female advisor should know the alien's taste better. Earlier research has indeed shown that gender stereotyping of tasks is manifested in the interaction among real humans (see Jeanquart-Barone and Sekaran, 1994; Eagly, 1997) and also in the interaction between a human and a robotic partner (see Kuchenbrandt et al., 2014; Tay et al., 2014). Thus, future research could investigate whether gender similarity (between user and robot) influences trusting beliefs and behavior when the task the robot has to perform is clearly related to gender stereotypes.

Likewise, this experiment found no effect of gender similarity on trusting behaviors (H2(b)), which is similar to our finding above regarding trusting beliefs (H1(b)). Furthermore, while male participants held higher trusting beliefs compared to female participants, female participants demonstrated higher trusting behaviors toward the advisor regardless of its gender asking the robot to make the selections on their behalf more often than men did. This finding is in agreement with an earlier study (Buchan et al., 2008) wherein an investment game with a similar decision structure male participants viewed the interaction more strategically than female participants in the human-human interaction. Also, in line with the findings of Buchan et al. (2008) in human-human interaction, both male and females participants showed more trusting behaviors when their interaction partner (in this study, the advisor) was female (compared to a male advisor).

Most importantly, the current results show (H2(a)) that facial characteristics of the robot influenced the participant's trusting behaviors. As the participants perceived the most trustworthy faced advisor as more intelligent than the least trustworthy face advisor (from the score of perceived intelligence), the participants who interacted with the advisor with the most trustworthy face were willing to take the risk of losing one extra credit by letting the advisor make the selections on behalf of them. In contrast, participants in the least trustworthy face condition preferred to save their credit by making their own prediction and to guess the right answer rather than trusting their advisor. Some earlier studies (Todorov et al., 2005; Ballew and Todorov, 2007) showed that judgments of competence from faces could affect human trusting behaviors. However, this earlier research did not model precisely which types of facial characteristics might invoke competency.

Although gender similarity was not shown to impact trust (measured by both trusting beliefs and trusting behaviors), our results (H3(b)) did show an influence of gender similarity on psychological reactance (negative cognitions). That is, participants experienced lower psychological reactance (especially negative cognitions) when interacting with a similar gender advisor than with an opposite gender advisor. These results are in line with the similarity-attraction hypothesis (Byrne, 1971) and with earlier studies which demonstrated similar-gender preference in human-robot interaction (Eyssel et al., 2012), especially for young children (Sandygulova and O'Hare, 2018) and in human-human interaction (Lalonde et al., 2006), especially for females (Lockwood, 2006). Surprisingly, female advisors caused higher psychological reactance than male advisors. In our study, both male and female advisors actually delivered the same advisory dialogues and used similar facial expressions in conveying exactly the same advice. Still, participants felt angrier and had more negative cognitions toward the female advisor than toward the male advisor. This study is the first to examine the influence of gender similarity on psychological reactance for human-robot interactions. Future research should examine whether this result can be replicated and what its causes.

In addition (providing evidence for H3(a)), results showed that facial characteristics of the advisor influenced the participant's psychological reactance. Participants felt higher reactance toward a robot with the least trustworthy facial characteristics compared to the one with the most trustworthy characteristics. This finding may have been because participants were more attracted to the most trustworthy advisor (see Figure 7). As highlighted by earlier research (Oosterhof and Todorov, 2009; Sacco and Hugenberg, 2009), the facial characteristics of the advisor in this experiment related to emotional expressions and social attributions. That is, the least trustworthy characteristics are associated with angry-looking faces while the most trustworthy characteristics of faces showing a positive emotion/mood (i.e., happy). Therefore, it could be that participants felt more reactant by having intense interactions with the robot featuring the least trustworthy facial characteristics.

Mediation analysis revealed that trusting beliefs were entirely driven by the liking rate toward the robot. The more the participants liked the robot, the more participants believed it could be trusted. In other words, the facial characteristics of the robot featuring the least trustworthy face caused participants to have less trusting beliefs due to the fact that the participants did not like the least trustworthy facial characteristics. In contrast, results provided no evidence that liking mediated the relationship between the robot's facial characteristics and the participant's trusting behaviors (e.g., asking for help). So, irrespective of whether the participants expressed like or dislike toward the robot, they frequently asked for its help only if they found it risky to make the selections themselves. In general, the decision to ask for help from the robot was affected only by the facial characteristics of the robot. The more trustworthy the robot's face, the more often participants requested its help. The third mediation analysis showed that liking was a full mediator for psychological reactance (feelings of anger and negative cognitions). This finding is similar to the role of liking as a mediator for the influence of robot facial characteristics on trusting beliefs. The psychological reactance was only triggered if the participants did not like the advisor. To sum up, liking the robotic advisor more caused higher trusting beliefs and triggered less psychological reactance, but did not affect trusting behaviors. Thus, our mediation analyses explain the negative correlation found in H4 between trusting beliefs and psychological reactance. It seems that if people like a robotic advisor, they believe it can be trusted resulting in lower psychological reactance, but this is not reflected in their trusting behaviors. The role of liking as a mediating factor needs to be further investigated in order to guide the design of persuasive robots that people can trust that will not make them feel psychological reactance.

### Design implication

Overall, this study provides insights into how persuasive robots should be designed. We learnt that people felt lower trust (both trusting beliefs and trusting behaviors) and experienced higher psychological reactance toward the robot with the least trustworthy face than the robot with the most trustworthy face. As we found that liking is a mediator for trusting beliefs and psychological reactance, it is essential for human-robot interaction designers to model likeable facial characteristics for social robots to elicit positive social responses (especially high trusting beliefs and low psychological reactance) from the user. Second, the persuasiveness of social robots can be enhanced using female robot. However, a drawback of using female robot as a persuader is that the female robot may also cause higher psychological reactance to the participants compare to the male robot.

### Limitations and future works

In our study, even if the participants react negatively during the interaction with the advisor, the advisor would not change its behavior as it is out of our research scope. We propose the future research might extend the current work by investigating how trust and psychological reactance toward a robot might be affected if the robot is more interactive responding directly to the user's emotion. Future research could also extend the current work by investigating the importance of robot gender (and similarity of robot gender with participants' gender) when the robot's task is gender-stereotyped. Moreover, future research could explore some strategies for enhancing likeability of robots as a means to enhance persuasion and trust, and could delve more into the discrepancy between trusting beliefs and behaviors, and how these influence the human-robot interaction in different application contexts. Importantly, future research using a similar experimental design as this study will help to validate and replicate the findings presented in this study (especially the exploratory analysis), and can also extend the current findings by adding new measures or manipulations.

## Conclusion

This study has made the following contributions; (1) we have shown how appropriate design of the facial characteristics of a robot can engender high trusting beliefs, high trusting behaviors, and invoke low psychological reactance in human-robot interaction, (2) we have illustrated how similarity in gender between users and the persuasive robot induces lower psychological reactance (lower negative cognitions about the robot) than interaction with a robot of the opposite gender, and (3) through mediation analyses we have found that liking of the robot (depending on its facial characteristics) is a full mediator for trusting beliefs and psychological reactance. Finally (4) we have found that lower psychological reactance was correlated with higher trusting beliefs, but not with trusting behaviors. From a practical standpoint, our results demonstrated that persuasion could be more effective and cause less reactance by designing facial characteristics of robots to match those known from interpersonal psychology to evoke trust in people, and by personalizing persuasive robots to match the gender of the user. Moreover, since liking has been shown to have a mediating role, it appears that a very generic mechanism for enhancing persuasiveness and reducing reactance is to design robots that will be more likeable, which could potentially be achieved by simpler means such as the static external appearance of the robot.

## Author contributions

All persons who meet authorship criteria are listed as authors, and all authors certify that they have participated sufficiently in work to take responsibility for the content, design, analysis, interpretation, writing, and/or critical revision of the manuscript. JH assisted AG developed the verbal and non-verbal cues for the robot. AG worked out in designing the concept and the interface of the decision-making game with help from EB and PM. All authors collaborated in designing human-subjects studies. AG carried out the experiment and performed the set of analyses under the guidance of JH. The manuscript was drafted by AG and edited by JH, EB, and PM for significant intellectual merit. All authors give approval of the final version to be published.

### Conflict of interest statement

The authors declare that the research was conducted in the absence of any commercial or financial relationships that could be construed as a potential conflict of interest.
